# Exergoeconomic Assessment of Solar Absorption and Absorption-Compression Hybrid Refrigeration in Building Cooling

**DOI:** 10.3390/e20020130

**Published:** 2018-02-17

**Authors:** Yue Jing, Zeyu Li, Liming Liu, Shengzi Lu

**Affiliations:** 1 School of Electric Power, South China University of Technology, Guangzhou 510640, China; 2 Guangdong Province Key Laboratory of High Efficient and Clean Energy Utilization, South China University of Technology, Guangzhou 510640, China; 3 Guangdong Province Engineering Research Center of High Efficient and Low Pollution Energy Conversion, South China University of Technology, Guangzhou 510640, China

**Keywords:** solar cooling, absorption-compression hybrid, exergoeconomics

## Abstract

The paper mainly deals with the match of solar refrigeration, i.e., solar/natural gas-driven absorption chiller (SNGDAC), solar vapor compression–absorption integrated refrigeration system with parallel configuration (SVCAIRSPC), and solar absorption-subcooled compression hybrid cooling system (SASCHCS), and building cooling based on the exergoeconomics. Three types of building cooling are considered: Type 1 is the single-story building, type 2 includes the two-story and three-story buildings, and type 3 is the multi-story buildings. Besides this, two Chinese cities, Guangzhou and Turpan, are taken into account as well. The product cost flow rate is employed as the primary decision variable. The result exhibits that SNGDAC is considered as a suitable solution for type 1 buildings in Turpan, owing to its negligible natural gas consumption and lowest product cost flow rate. SVCAIRSPC is more applicable for type 2 buildings in Turpan because of its higher actual cooling capacity of absorption subsystem and lower fuel and product cost flow rate. Additionally, SASCHCS shows the most extensive cost-effectiveness, namely, its exergy destruction and product cost flow rate are both the lowest when used in all types of buildings in Guangzhou or type 3 buildings in Turpan. This paper is helpful to promote the application of solar cooling.

## 1. Introduction

Solar absorption chillers present potential for utilizing sustainable fuels to reduce air conditioning energy consumption and greenhouse gas emission [1]. The fact that collectors are usually installed at the roof of buildings leads to that the maximum cooling output of an individual solar absorption chiller cannot meet the entire building cooling demand, especially for the high-rise buildings [2]. Since natural gas is cheaper and cleaner than oil, solar/natural gas-driven absorption chiller (SNGDAC) is commonly used to increase the total cooling output. Although its investment cost increment mainly coming from the auxiliary heat machine is little compare with single-effect solar absorption chiller, its operational cost increment caused by the consumption of natural gas is high especially when applied on the high-rise buildings [3]. Therefore, the economic performance of SNGDAC is determined by the quantity of natural gas consumption, and moreover, the scale of buildings.

Besides this, types of compression–absorption hybrid systems were put forward recently, such as solar vapor compression–absorption integrated refrigeration system with parallel configuration (SVCAIRSPC) [4] and solar absorption-subcooled compression hybrid cooling system (SASCHCS) [5]. Compared with SNGDAC, the operational cost of compression–absorption hybrid system is lower but the investment cost is higher. In SVCAIRSPC, the chilled water flows through the evaporators of the absorption subsystem and compression subsystem successively to output the cooling. However, in SASCHCS, the cooling output of absorption subsystem does not directly cool the chilled water but subcools the refrigerant of the compression subsystem. Consequently, not only can its exergy destruction of throttling valve in the compression chiller be decreased, but its evaporator temperature of the absorption chiller can also be improved compared with SVCAIRSPC [5]. Nevertheless, the advantage of SASCHCS becomes weaker with the growth of cooling capacity in the absorption subsystem because of the significant decline of evaporator temperature in the absorption subsystem [6]. Even the evaporator temperature of the absorption subsystem can be lower than 0 °C only if the size of absorption chiller is great enough. In this case, the thermodynamic performance of SASCHCS is obviously poorer than SVCAIRSPC. To sum up, the strength of SNGDAC benefits to its lower investment cost compared with the other two hybrid systems, and for the two hybrid systems, which configuration has the better thermodynamic performance depends on the required cooling output of the absorption subsystem. Consequently, in order to provide a suitable solar absorption cooling for those buildings requiring different cooling demands and located under different solar irradiance, it is essential to compare the performance of the above three configurations based on a proper method in which the trade-off of system investment and operation cost is contained.

The exergoeconomic analysis combining both thermodynamics and economics is helpful to perform the comprehensive comparison of the above three configurations [7]. In exergoeconomics, exergy is used instead of energy as thermodynamic criteria to analysis the energy transfer in different components [8]. The exergoeconomic analysis has been used in many absorption refrigeration systems. Preliminarily, this method was applied to the optimization of a single-effect LiBr–H_2_O absorption system, aiming at minimizing its overall operation and amortization cost [9]. In addition, exergoeconomic analysis was used to design a phase-change material (PCM) storage system of single-effect absorption chiller [10], but the result showed that the payback period increases caused by the auxiliary storage system. Subsequently, a double-effect LiBr–H_2_O absorption system was optimized by a simplified cost minimization methodology based on exergoeconomics [11]. In order to investigate the effect of different thermodynamic parameters on thermoeconomic performance, a deeper exergoeconomic analysis of three types of LiBr–H_2_O double-effect absorption refrigeration systems were performed [12]. Besides this, a combined ejector double-effect system was also included [13]. In that research, series flow double-effect system and combined ejector double-effect system were compared, and the influences of various operating parameters on investment cost and product cost flow rate were analyzed. The result showed that the combined cycle operates more economically than the double effect system. In addition, this method was extended to an NH_3_–H_2_O vapor-absorption refrigeration system [14]. A contrast study of NH_3_–H_2_O absorption chiller and LiBr–H_2_O absorption chiller was done from the exergoeconomic viewpoint [15]. It was shown that the NH_3_–H_2_O absorption chiller had the highest exergy destruction cost rate, and the LiBr–H_2_O absorption chiller had the lowest one.

The exergoeconomic analysis was also used to the hybrid refrigeration systems. For a cascade absorption–compression refrigeration system, cooling set and solution heat exchanger should be designed carefully according to the exergoeconomic factor values [16]. And the multi-objective optimization of a cascade hybrid system was performed based on the NSGA-II technique, which combines both thermodynamics and total product cost flow rate [17]. For hybrid generator–absorber heat exchange (GAX) refrigeration absorption cycle, its coefficient of performance (COP) and exergy efficiency was higher than standard GAX cycle, but its product cost per exergy unit was higher, too [18]. Also, it was found that the cooling capacity of the absorption subsystem, the condenser_2_ temperature, the evaporator temperature, and the isentropic efficiency of compressor in the absorption-subcooled compression hybrid cooling system should be designed carefully from the exergoeconomic viewpoint [6]. Apart from refrigeration systems, exergoeconomics was also used to analysis multi-effect evaporation-absorption heat pump and vapor-compression refrigeration [19]. It was found that the system with two refrigerant compressors was flexible in order to allocate different energy sources, and the parameter optimizations were done later [20]. Besides this, the exergoeconomic analysis was used to reduce the fuel cost flow rate and the exergy destruction cost of a novel solar-trigeneration system for heating, cooling, and power production [21]. Also, the exergoeconomic analysis based on a novel combined SCRB–ARC (supercritical CO_2_ recompression Brayton–absorption refrigeration cycle) showed that the largest exergy destruction rate belongs to the reactor, while the components in the ARC have less exergy destruction [22]. Furthermore, the comprehensive exergoeconomic comparison of three types of absorption power and cooling cogeneration cycles based on Kalina revealed that the first configuration of simple ammonia-water absorption refrigeration/Kalina co- generation cycle has the lowest cost of products and payback period [23]. A novel solar-based combined system containing a concentrated photovoltaic thermal (CPVT), a single effect LiBr–H_2_O absorption chiller, and proton exchange membrane electrolyzer (PEM) was also analyzed in detail by exergoeconomics, and it was found that the maximum exergy destruction occurs in the CPVT followed by absorption chiller unit and PEM electrolyzer [24].

SNGDAC, SVCAIRSPC, and SASCHCS are three typical promising solar absorption chillers to solve building cooling. However, the optimal match of above-mentioned facility and different types of buildings based on a comprehensive consideration of thermodynamic and economic performance is still not clear. It was found that exergoeconomics is a powerful method to present the exergoeconomic comparison of SNGDAC, SVCAIRSPC, and SASCHCS thermodynamically and economically. Consequently, this paper aims to obtain the appropriate solution for different types of buildings cooling based on the exergoeconomic comparison. Three types of buildings and two Chinese cities with different solar irradiance, Guangzhou and Turpan, are taken into account. The product cost flow rate is employed as the primary decision variable. Besides, the match of three solar refrigeration systems and building cooling is analyzed and explained thoroughly by the exergy and cost. The novelty of paper is the presentation of appropriate solar refrigeration system for different types of building cooling. The paper is helpful to promote the application of solar cooling.

## 2. System Description

In each solar absorption refrigeration system, the solar system consists of the evacuated tube collectors (ETC) and a hot water storage tank. The working fluids of collectors, absorption chillers, and compression chillers are pressurized water, LiBr–H_2_O, and R410A, respectively. Besides this, the cooling capacity of absorption chillers in SVCAIRSPC and SASCHCS are restricted by both the installation area of collectors and the intensity of solar irradiance. Several structural features of three systems are described as follows:

- Solar/Natural Gas-Driven Absorption Chiller (SNGDAC)

**Figure 1:**
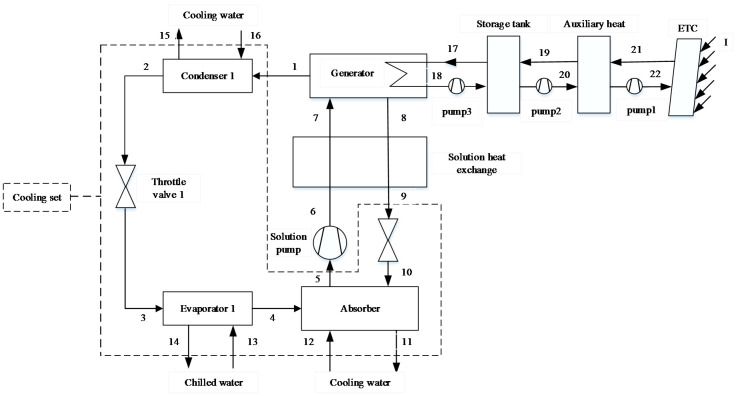
Schematic of Solar/Natural Gas-Driven Absorption Chiller (SNGDAC).

The schematic of SNGDAC is shown in Figure 1. This plant consists of a solar system, an auxiliary heat system, a single-effect absorption chiller equipped with a cooling tower, liquid pipelines, and valves. In this machine, a natural gas burner is used as auxiliary device in case that the cooling output of absorption chiller cannot satisfy the maximum cooling demand of buildings. Moreover, when the solar irradiance cannot meet the required demand of generator, the auxiliary heater is switched on to fulfill the entire energy requirement of absorption chiller.

- Solar Vapor Compression–Absorption Integrated Refrigeration System with Parallel Configuration (SVCAIRSPC)

**Figure 2:**
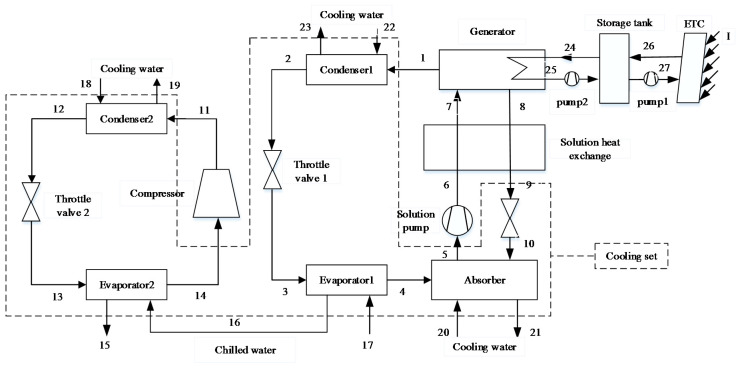
Schematic of Solar Vapor Compression–Absorption Integrated Refrigeration System with Parallel Configuration (SVCAIRSPC).

The schematic of SVCAIRSPC is shown in Figure 2. This plant consists of a solar system, a single-effect absorption chiller, a vapor compression chiller, liquid pipelines, and valves. In SVCAIRSPC, the cooling load of buildings are shared by two subsystems, and the chilled water is cooled in the evaporators of absorption subsystem and compression subsystem successively [4].

- Solar Absorption-subcooled Compression Hybrid Cooling System (SASCHCS)

**Figure 3:**
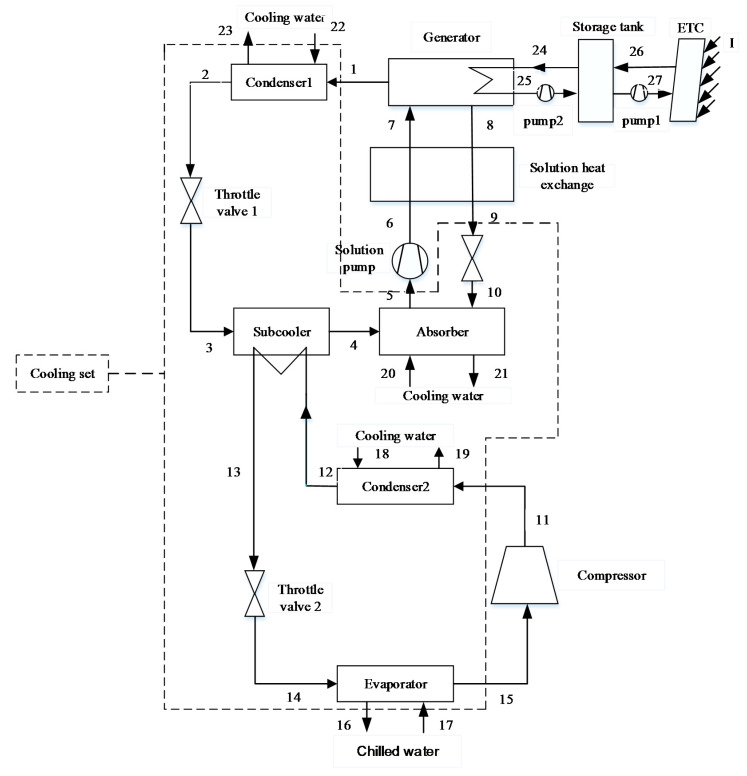
Schematic of Solar Absorption-Subcooled Compression Hybrid Cooling System (SASCHCS).

The schematic of SASCHCS is shown in Figure 3. Similar with SVCAIRSPC, this machine consists of an absorption section and a compression section. Nevertheless, the evaporator of absorption section also works as the subcooler of the compression section. That is, the cooling capacity of the absorption chiller directly subcools the refrigerant of the compression subsystem instead of cooling the chilled water. Consequently, the exergy destruction of the throttling valve in the compression section is decreased and the consumption of the compressor is saved [5].

## 3. Building Description

The buildings simulated by Hongye program [25] in this paper are divided into three types: type 1 is the single-story building, type 2 includes the two-story and three-story buildings, and type 3 is the multi-story building (its number of floor is from four to ten). The design parameters of three types of buildings are the same except the number of floors. The area of each floor is 360 m^2^ containing ten rooms, and the daily ventilation rate is taken as 30 m^3^/h/person. The window-to-wall ratio equals to 0.3 (the windows are uniformly distributed on the lateral surfaces). In the whole operational period, the room temperature is controlled between 22 °C and 26 °C. In particular, the cooling ventilation is considered when the outdoor surrounding temperature is lower than 22 °C. The heat transfer coefficient of windows, doors, walls, and roofs are 2.7 W/(m^2^·K), 2.7 W/(m^2^·K), 1.081 W/(m^2^·K), and 0.812 W/(m^2^·K), respectively.

## 4. Model

### 4.1. Thermodynamic Model

The thermodynamic models of three solar absorption systems are based on following assumptions:

- The systems are operated under steady state.
- The states of working fluid at the exit of condensers, evaporators, and absorbers are saturated.
- The pressure and heat loss in pipelines and components are neglected.
- The components of cooling systems are adiabatic.
- The cooling water is 25 °C and 100 kPa.

The balances of mass and energy conservation are formulated as follows:

(1) $$\mathrm{Mass}\;\mathrm{balance}:\sum m_{i}=\sum m_{o}$$

(2) $$\mathrm{Material}\;\mathrm{balance}:\sum m_{i}x_{i}=\sum m_{o}x_{o}$$

(3) $$\mathrm{Energy}\;\mathrm{balance}:Q=\sum m_{i}h_{i}-\sum m_{o}h_{o}.$$

The efficiency of ETC is evaluated as follows [26]:

(4) $$\eta_{etc}=0.612-1.98(T_{i}-T_{a})/I.$$

Prior to calculating the heat transfer coefficient, the types of heat exchangers are introduced. The generator is a falling film vertical tube heat exchanger. In generator, the hot water and the LiBr–H_2_O solution fluid flow inside and outside the tubes respectively. The condensers are taken as the horizontal tube heat exchanger. The R410A–H_2_O is condensed on the outside surface of the tubes and the cooling water flows inside the tubes. The absorber is a falling film vertical tube heat exchanger. The solution flows downward on the outside surface of the tubes so that the water vapor from the evaporator can be absorbed totally, and the cooling water flows inside the tubes. The evaporator is a horizontal tube heat exchanger. The working fluid (R410A–H_2_O) is evaporated on the outside surface of the tubes and the chilled water flows inside the tubes. The subcooler in SASCHCS is a falling film horizontal tube heat exchanger. The water vapor is evaporated on the outside surface of the tubes and the refrigerant (R410A) flows inside the tubes. Besides this, the solution heat exchanger is a double-pipe heat exchanger. The strong LiBr solution flows inside the inner pipes, and the weak LiBr solution flows outside the pipes.

The correlations referred to the calculation of the heat transfer coefficients in the heat exchanges are summarized in Table 1. Besides, the inner diameter, the thickness, the length, and the fouling factors of tubes are taken as 25 mm, 2 mm, 1 m, and 0.09 m^2^·°C/kW, respectively [13].

The area of heat exchange is calculated as:

(5) $$A=\frac{Q}{U*LMTD}.$$

*U* is the heat transfer coefficient of overall system:

(6) $$U=\frac{1}{\frac{1}{h_{o}}+F_{o}+\frac{d_{o}}{d_{i}}F_{i}+\frac{d_{o}}{d_{i}}\frac{1}{h_{i}}}.$$

*LMTD* is the mean temperature difference between the hot and the cold fluid that is expressed as:

(7) $$LMTD=\frac{\Delta T_{0}-\Delta T_{L}}{\ln\frac{\Delta T_{0}}{\Delta T_{L}}}.$$

### 4.2. Exergoeconomic Model

Prior to exergoeconomic analysis, it is essential to establish the exergy model. In order to analyze the performance of a system from the second law point of view, the Fuel-Product-Loss (F-P-L) model of exergy flow rate is established. The fuel represents the exergy input to obtain the product and is not restricted into actual energy such as solar or natural gas [33]. The product represents the required result of components or total systems. Based on the F-P-L model, SNGDAC is divided into the following components: collectors, store tank, auxiliary heat device, generator, solution heat exchange, pump, and cooling set (enclosed by dash line in Figure 1). The other two hybrid systems are divided into collectors, store tank, generator, solution heat exchange, pump, compressor, and cooling set (enclosed by dash line in Figure 2 and Figure 3). The definitions of the fuel, product, and loss of exergy for each component are summarized in Table 2.

The exergy of each point is calculated as:

(8) $$E=me=m\left[\left(h-h_{0})-T_{0}(s-s_{0}\right)\right].$$

The exergy destruction is calculated by the exergy balances of the components:

(9) $$E_{D}=\sum E_{i}-\sum E_{o}.$$

Besides, the exergy destruction can be calculated based on the F-P-L model as well:

(10) $$E_{D}=E_{F}-E_{P}-E_{L}.$$

To calculate the investment costs of heat exchange, solution pump and compressor, a power law related is used, such as [34]:

(11) $$Z_{HE}=Z_{R,HE}(\frac{A}{A_{R,HE}}{)}^{\alpha }$$

(12) $$Z_{SP}=Z_{R,SP}\left(\frac{W_{SP}}{W_{R,SP}}\right){}^{m1}{\left(\frac{1-\eta_{sp}}{\eta_{sp}}\right)}^{n1}$$

(13) $$Z_{COM}=Z_{R,COM}{\left(\frac{W_{COM}}{W_{R,COM}}\right)}^{m1}{\left(\frac{\eta_{s,COM}}{0.9-\eta_{s,COM}}\right)}^{n2}$$

Here, the powers in the equations take on the following values:

*α* = 0.6, *m*_1_ = 0.26, *n*_1_ = 0.5, *m*_2_ = 1.0, *n*_2_ = 0.5

The other values of corresponding parameters are listed on Table 3.

In order to update the investment cost to year 2018, the chemical engineering plant cost index (CEPCI) [35] is defined as follow:

(14) $$Z=Z_{\mathrm{oy}}\frac{CEPCI}{CEPCI_{\mathrm{oy}}}.$$

In order to convert the investment cost of a component into the cost flow rate, the levelized investment cost is gotten by the multiplication of capital investment cost and capital recovery factor (CRF). The CRF is determined as follows:

(15) $$CRF=\frac{i{\left(1+i\right)}^{N}}{{\left(1+i\right)}^{N}-1}.$$

Here, *i* represents the 2018 Chinese interest rate and is equal to 0.0435. *N* is the operational period of systems and it is considered as 20 years [13].

In order to calculate the cost flow rate of each working fluid, it is essential to formulate some equations of component based on the F-P-L model. The corresponding auxiliary equations are shown in Table 4. The exergoeconomic analysis is mainly based on the fuel cost per exergy unit, the product cost per exergy unit, the exergy destruction cost flow rate, and the loss cost flow rate. The corresponding parameters are defined as follows:

(16) $$c_{F}=C_{F}/E_{F}$$

(17) $$c_{P}=C_{P}/E_{P}$$

(18) $$C_{D}=c_{F}E_{D}$$

(19) $$C_{L}=c_{F}E_{L}$$

### 4.3. Model Validation and Case Study

**Figure 4:**
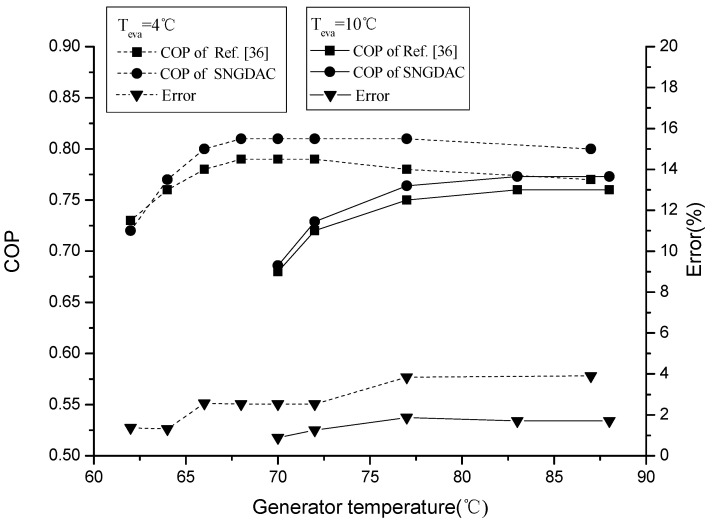
Comparison of COP of SNGDAC with Reference [36].

In order to validate the thermodynamic model of SNGDAC, the experimental results reported by Reference [36] are considered. The input data are *T_cond_* = 33 °C, *T_a_* = 33 °C, *T_eva_* = 4/10 °C, and *ε_shx_* = 0.7. The comparison of COP of SVCAIRSPC with Reference [33] is shown in Figure 4. It was found that the error in the computation of COP is less than 3.89%.

The SVCAIRSPC is validated with corresponding numerical results presented by Reference [4]. The following input data are used for comparison: *Q_e_* = 170 kW, *T_cond_* = 37 °C, *T_g_* = 90 °C, *T_a_* = 37 °C, *T_eva-a_* = 6 °C, and *T_eva-c_* = 1 °C. The comparison of performance data of SVCAIRSPC with Reference [4] is shown in Table 5. It was found that the error in the calculated values is less than 2.48%.

Besides this, the validation of SASCHCS has been done in our previous research [6].

A case study associated with the exergy and exergoeconomic analysis of SNGDAC, SVCAIRSPC, and SASCHCS is carried out. The corresponding design conditions are shown in Table 6. The meteorological data of the base case is collected from the subtropical Guangzhou [5,6]. The annual operational period of air conditioning is from April to October and assumed to be 2100 h. The mean solar irradiance and the average surrounding temperature is 500 W/m^2^ and 30.2 °C [6]. Calculated by Hongye program, the average daily cooling demand of this building is 158 kW. The unit prices of electric energy, storage tank, and ETC are considered as 0.136 $/kWh, 234.45 $/m^3^, and 93.8 $/m^2^ based on the Chinese current price. In addition, the exergoeconomic models are established by FORTRAN program. The thermodynamic parameters of working fluids are calculated by the correlation of Pάtek and Klomfar [37] and the software Refprop9 [38].

According to the distribution of solar energy resources in China released by the China Association for Science and Technology [39], the summer cooling demand of Guangzhou is great but its summer solar irradiance is weak, and Turpan is the region with strong summer cooling demand as well as solar irradiance. Consequently, in the further research of this paper, we selected Guangzhou and Turpan as the sources of case meteorological parameters to study the comparison of the cost effectiveness of SNGDAC, SVCAIRSPC, and SASCHCS in different regions. Besides this, the summer average cooling demands of type 1 to type 3 buildings are contained to obtain the better cost effective solar absorption chillers for different types of buildings. The summer average cooling demands of office buildings located in Guangzhou and Turpan are shown in Table 7.

## 5. Results and Discussions

### 5.1. Comparison Based on Exergy and Exergoeconomics Analysis

The state property of each point in three chillers is listed in Table 8, and the results of exergy analysis of SNGDAC, SVCAIRSPC, and SASCHCS are demonstrated in Table 9. According to Table 9, the maximum value of *E_D_* in SNGDAC belongs to the cooling set involving 47.63% of $E_{D}^{tot}$, and the generator and the solution heat exchanger are in the next ranking containing 28.85% and 23.52% of $E_{D}^{tot}$, respectively. For the other two hybrid systems, the cooling sets in both SVCAIRSPC with 51.71% of $E_{D}^{tot}$ and SASCHCS with 53.80% of $E_{D}^{tot}$ are dominant. The exergy efficiency of SASCHCS is the highest (23%) and that of SNGDAC is the lowest (17%) in the base case. Besides this, the total system fuel exergy of SNGDAC and SVCAIRSPC is 32.94% and 12.25% higher than SASCHCS, respectively, to produce the same cooling power.

Further results obtained from the exergoeconomic analysis are listed in Table 10. It implies that the cooling sets of SNGDAC have the maximum $C_{D}+C_{L}+Z$ with 69.95% of $C_{D}^{tot}+C_{L}^{tot}+Z^{tot}$, followed by the generator and the solution heat exchanger containing 15.63% and 13.78% of $C_{D}^{tot}+C_{L}^{tot}+Z^{tot}$. The cooling sets in both SVCAIRSPC with 71.80% of $C_{D}^{tot}+C_{L}^{tot}+Z^{tot}$ and SASCHCS with 70.91% of $C_{D}^{tot}+C_{L}^{tot}+Z^{tot}$ are remarkable. It is obvious that SNGDAC has the highest $C_{D}^{tot}+C_{L}^{tot}+Z^{tot}$ and SASCHCS has the lowest one. Besides, both the $c_{F}^{tot}$ and $c_{P}^{tot}$ of SNGDAC are obviously the highest, and the $c_{F}^{tot}$ of SVCAIRSPC and SASCHCS are almost the same while the $c_{P}^{tot}$ of SASCHCS is slightly lower. As shown in Table 10, in the base case, the r of SNGDAC is the lowest, owing to the smaller difference between its $c_{F}^{tot}$ and $c_{P}^{tot}$, and the r of SVCAIRSPC and SASCHCS are 67.72% and 18.99% higher, respectively, than that of SNGDAC.

### 5.2. Comparison Based on Guangzhou

The product cost flow rate obtained simply by replacing the exergy rate by cost rate is an important criterion to evaluate the cost-effectiveness of a system. According to chapter 8 in Reference [33], the product cost flow rate is strongly influenced by the fuel cost flow rate and the exergy efficiency or the exergy destruction. Accordingly, this section mainly compares the total product cost flow rates of three types of absorption chillers, i.e., SNGDAC, SVCAIRSPC, and SASCHCS, to determine the best cost-effective system in different conditions, and besides this, the total fuel cost flow rate and the total exergy destruction are taken as the auxiliary criteria to understand the cost-effectiveness of the three chillers.

**Figure 5:**
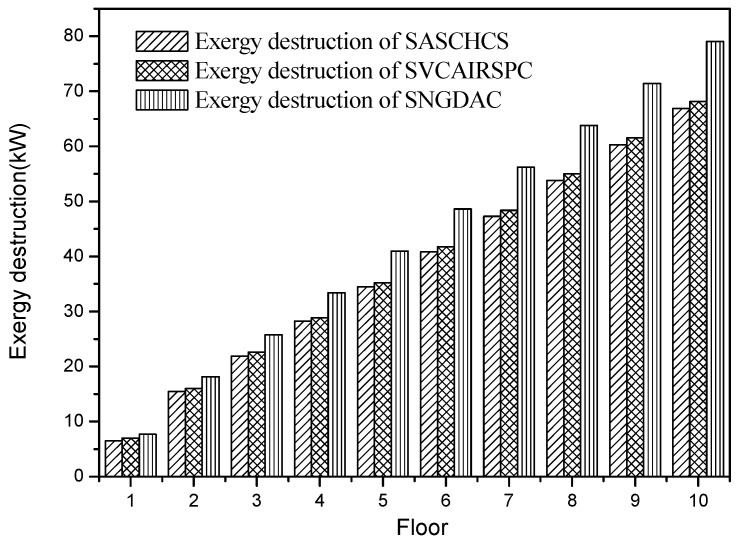
Exergy destruction based on Guangzhou.

The exergy destruction of the three chillers used in three types of buildings located in Guangzhou is shown in Figure 5. It is demonstrated that SASCHCS has the lowest exergy destruction and SNGDAC has the highest one, and such result of comparison is independent to the number of floors of the buildings. It can be explained by that the compressor in the vapor compression chiller is replaced by the generator, the solution heat exchange, and the absorber, but the thermodynamic efficiency of the compressor is obviously better than such replacement. Consequently, the exergy destruction of SNGDAC is around 17%–19% higher than SASCHCS and 13%–16% higher than SVCAIRSPC for all types of buildings. Besides this, in SASCHCS, the cooling output of the absorption subsystem does not directly cool the chilled water but subcools the refrigerant of compression subsystem to decrease the input enthalpy of the throttling valve. Therefore, its exergy destruction of the throttling valve in the compression subsystem is decreased, which results in that the exergy destruction of total system in SASCHCS is less than that in SVCAIRSPC.

**Figure 6:**
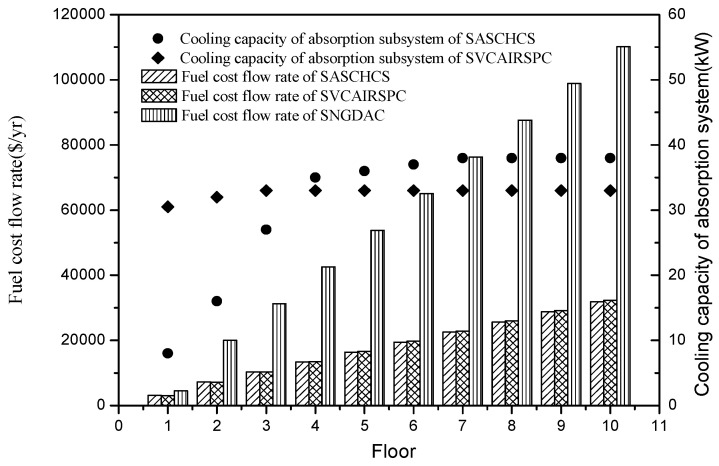
Fuel cost flow rate and cooling capacity of the absorption system based on Guangzhou.

**Figure 7:**
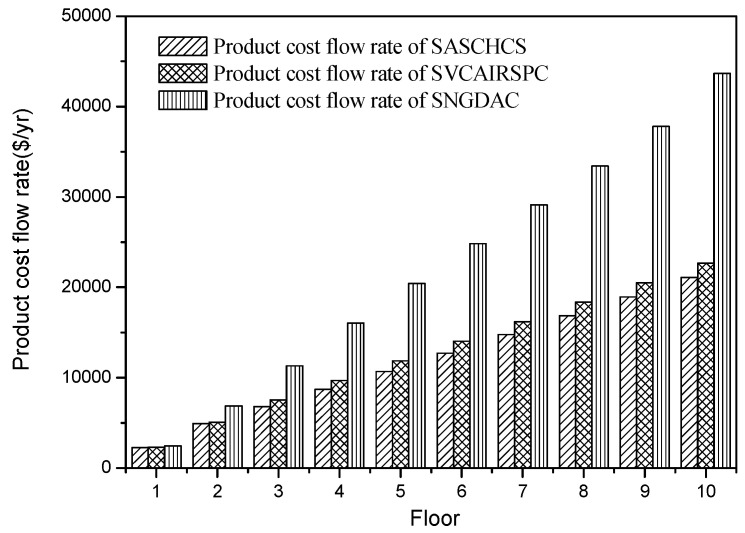
Product cost flow rate based on Guangzhou.

The total fuel cost flow rates of the three chillers and the cooling capacities of the absorption subsystem of the two hybrid chillers are shown in Figure 6. It was found that the fuel cost flow rate of SNGDAC is always the highest for the single-story to ten-story office buildings located in Guangzhou. That is attributed to its low COP caused by the large natural gas consumption. Besides this, based on the fundaments of exergoeconomics, the total fuel cost flow rate of a solar absorption–compression hybrid system is mainly derived from the investment cost of the solar system and the consumption of the compressor. In this section, the cooling capacity of the absorption subsystem in the two hybrid systems is restricted by both the maximum installation area of collectors (cannot exceed 270 m^2^) and the evaporator temperature in the absorption chiller (cannot be below 4 °C). For the hybrid systems applied to the low-rise buildings, the decline of evaporator temperature in absorption chiller with the rise of cooling capacity of absorption subsystem in SASCHCS is much more notable than the decline in SVCAIRSPC. Therefore, as shown in Figure 7, the actual cooling capacity of the absorption subsystem in SASCHCS is obviously lower than that in SVCAIRSPC for the type 1 and type 2 buildings, and its compressor consumption and total fuel cost flow rate is higher. However, for SASCHCS, the limit of evaporator temperature in absorption chiller on the cooling capacity of absorption becomes negligible when used in the high-rise buildings. Meanwhile, the evaporator temperature in absorption chiller of SASCHCS is even higher than that of SVCAIRSPC because of its better thermodynamic performance [5]. Accordingly, the actual cooling capacity of absorption subsystem in SASCHCS is slightly higher than that in SVCAIRSPC when applied to the type 3 buildings, as observed in Figure 7, and its compressor consumption and total fuel cost flow rate are lower. However, the difference of the fuel cost flow rate of SASCHCS and SVCAIRSPC is within ±3% for all three types of buildings.

The results of the comparison of three chillers based on the total product cost flow rate are shown in Figure 7. It is seen that the product cost flow rate of SASCHCS is the least for all three types buildings (1–10 floors) located in Guangzhou. That is attributed to its obviously lowest exergy destruction even though its fuel cost flow rate for the type 1 and type 2 buildings is slightly higher than SVCAIRSPC. Besides this, the product cost flow rate of SNGDAC is always the highest because of its highest exergy destruction as well as fuel cost flow rate. Such product cost flow rate is within 10% higher for the single-story building, but even exceeds 90% higher when the number of floors exceeds eight.

### 5.3. Comparison Based on Turpan

**Figure 8:**
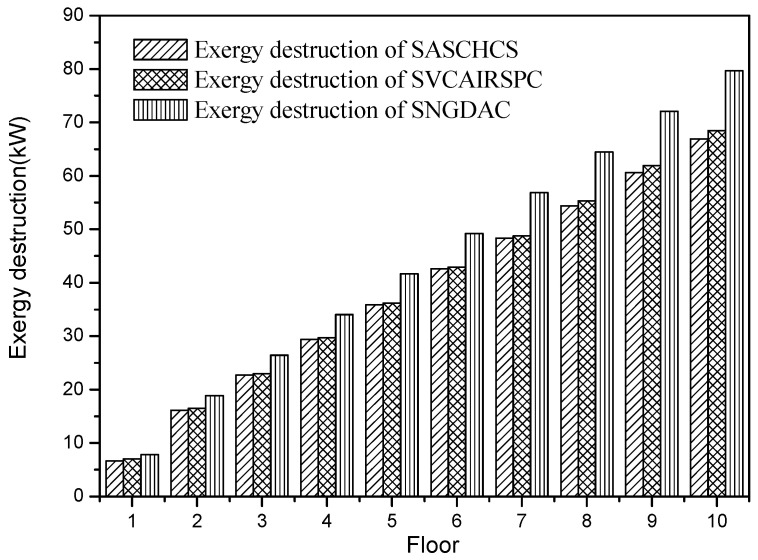
Exergy destruction based on Turpan.

The total exergy destruction of the three chillers for the types of buildings located in Turpan is shown in Figure 8. It was found that the result of comparison based on the exergy destruction is independent of the region (or the solar irradiance) as well as type of building. That is, the SNGDAC has the highest exergy destruction while the SASCHCS has the lowest one for all buildings, and the excessive exergy destruction of SNGDAC is around 12%–19% higher than the other two hybrid systems. 

**Figure 9:**
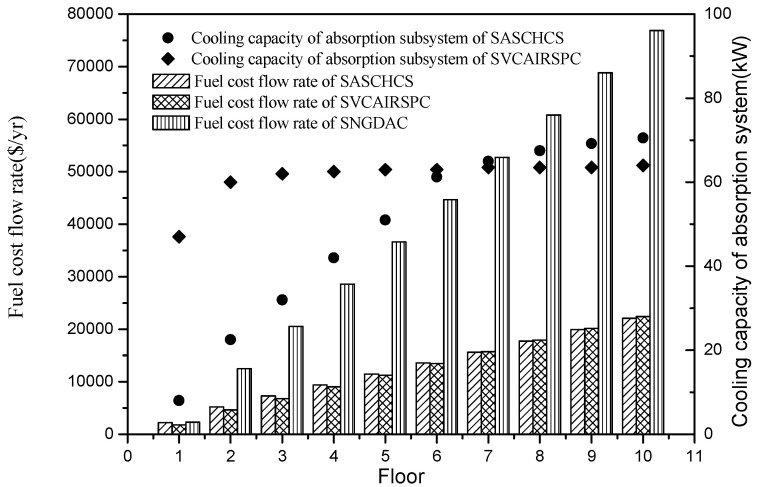
Fuel cost flow rate and cooling capacity of absorption system based on Turpan.

**Figure 10:**
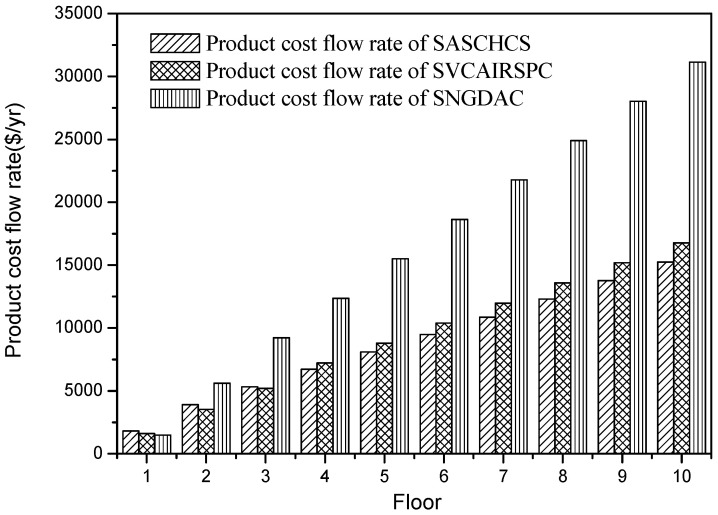
Product cost flow rate based on Turpan.

The total fuel cost flow rates of the three chillers and the cooling capacities of the absorption subsystem of the two hybrid chillers are shown in Figure 9. According to Reference [39], the summer mean solar irradiance of Turpan (around 780 W/m^2^) is stronger than that of Guangzhou (around 500 W/m^2^), which results in its higher efficiency of collectors. As a result, in the absorption subsystem of SASCHCS, the limit of evaporator temperature on its actual cooling output is more obvious. That leads to the result that for the single-story to six-story buildings, the actual cooling capacities of absorption subsystem in SASCHCS are lower than those in SVCAIRSPC, and consequently its total fuel cost flow rates are higher, as shown in Figure 10. Besides, the total fuel cost flow rate of SASCHCS is slightly higher than that of SVCAIRSPC for the buildings that exceed six floors because of its higher cooling capacity of the absorption subsystem, which is similar with the result of comparison for buildings that exceed three floors located in Guangzhou. However, the difference of total fuel cost flow rate of SASCHCS and SVCAIRSPC is negligible when the number of floors exceeds four. In addition, the total fuel cost flow rate of SNGDAC is still the highest regardless of the number of floors or the intensity of local mean solar irradiance, and such superfluity becomes significant especially for the mid-rise and high-rise buildings.

As shown in Table 7, the cooling demand of a type 1 building located in Turpan is about 48 kW. It is obvious that the cooling output of a single-effect absorption chiller with 270 m^2^ collectors can almost meet its cooling demand. In this case, for the type 1 buildings, the natural gas consumption of SNGDAC is negligible and its total fuel cost per exergy unit mainly depends on the investment cost of solar collectors. Meanwhile, the fuel costs per exergy unit of SASCHCS or SVCAIRSPC are determined by both the investment cost of solar collectors and the consumption of the compressor. It has been found that the fuel cost per exergy unit that relies on the investment cost of solar collectors is less than that which relies on the consumption of the compressor [13]. Consequently, for the type 1 buildings located in Turpan, the fuel cost per exergy unit of SNGDAC is less than that of SASCHCS or SVCAIRSPC (Figure 10). Actually, the fuel costs per exergy unit of SNGDAC, SASCHCS, and SVCAIRSPC used in the type 1 buildings located in Turpan are 30.559 $/GJ, 34.748 $/GJ, and 33.457 $/GJ, respectively in this paper. Furthermore, the low fuel cost per exergy unit of SNGDAC leads to the low product cost per exergy unit as well as product cost flow rate when applied to the type 1 buildings, as shown in Figure 10. Besides this, for the type 2 buildings, the product cost flow rate of SVCAIRSPC is the lowest across the three chillers due to its lowest fuel cost flow rate. Moreover, SASCHCS has the lowest product cost flow rate because of its lowest exergy destruction when used in the four-story to six-story buildings located in Turpan, even though its fuel cost flow rate is slightly higher. For the office buildings above six floors, SASCHCS still has the lowest product cost flow rate, and its lowest fuel cost flow rate and exergy destruction are responsible for that. Namely, SASCHCS is more cost-effective for all type 3 buildings located in Turpan, and its total product cost flow rate is around 9% and 50% lower than SVCAIRSPC and SNGDAC, respectively. 

## 6. Conclusions

In this paper, the exergoeconomic evaluation of SNGDAC, SVCAIRSPC, and SASCHCS for different types of buildings cooling is performed. Three types of buildings cooling and two Chinese cities, Guangzhou and Turpan, are considered. The corresponding conclusions are summarized as follows:

1. For all three types of buildings (1–10 floors) located in Guangzhou, SASCHCS shows the best cost-effectiveness, owing to its lowest total fuel and product cost flow rate as well as the least exergy destruction.
2. For the type 1 buildings (1 floor) located in Turpan, SNGDAC is considered as a suitable solution because of its negligible natural gas consumption and lowest total product cost flow rate.
3. For the type 2 buildings (2–3 floors) located in Turpan, SVCAIRSPC is more cost effective than the others, and that is attributed to its higher actual cooling capacity of absorption subsystem and the lowest fuel and product cost flow rate.
4. For the type 3 buildings (4–10 floors) located in Turpan, SASCHCS has the lowest exergy destruction and product cost flow rate, namely, the optimum cost effectiveness.

## Figures and Tables

**Table 1:** Heat transfer coefficients.

Component	Application	Heat Transfer Coefficient
Generator	Inside [27]	h=129.7712xi−0.8058qw0.2422Re−0.0856
	Outside [28]	Nu=0.348εnRe0.592
Condenser	Inside [13]	$\left\{ \begin{array}{l} h=0.555{\left[\frac{g\rho_{l}\left(\rho_{l}-\rho_{v}\right)\lambda_{l}^{3}r^{*}}{\mu_{l}\left(T_{sat}-T_{w}\right)}\right]}^{\frac{1}{4}}\\ r*=r+\frac{3}{8}c_{p,l}(T_{sat}-T_{w}) \end{array}\right.$
	Outside	Similar to the generator
Absorber	Inside [29]	Nu=0.023Re0.8Prn
	Outside [30]	{h=λδ0.029Re0.53Pr0.344δ=(3μΓ/ρ2g)1/3Re=4Γ/μΓ=m/μd
Evaporator	Inside [31]	{h=Fhl+ShpoolF=1+(1/X)1.051+Weg−0.4S=11+0.4(Re×10−4)1/4hpool=207λldb(qdbλlTl)0.745(ρvρl)0.581Prl0.533db=0.51[2σ/g(ρl−ρv)]0.5
	Outside	Similar to the generator
Subcooler	Inside	Similar to the absorber
	Outside [32]	Nu=0.041Re0.03PrlAr−0.04
Solution heat exchange [29]		Nu=0.012(Re0.87−280)Pr0.4(1+d/l)2/3(Pr/Prw)0.11

**Table 2:** Definition of fuel, product, and loss exergy flow rate of the three systems.

Component	Fuel	Product	Loss
**SNGDAC**			
Collector	*I*	*E*_21_ − *E*_22_	-
Auxiliary heat	*E*_21_ − *E*_22_	*E*_19_ − *E*_20_	-
Storage tank	*E*_19_ − *E*_20_	*E*_17_ − *E*_18_	
Generator	*E*_17_ − *E*_18_	*E*_1_ + *E*_8_ − *E*_7_	-
Solution heat exchange	*E*_8_ − *E*_9_	*E*_7_ − *E*_6_	-
Pump	*W_sp_*	*E*_6_ − *E*_5_	-
Cooling set	*E*_1_ + *E*_9_ − *E*_5_	*E*_14_ − *E*_13_	*E*_15_ − *E*_16_ + *E*_11_ − *E*_12_
Overall cooling system	*E*_17_ − *E*_18_ + *W_sp_*	*E*_14_ − *E*_13_	*E*_15_ − *E*_16_ + *E*_11_ − *E*_12_
**SVCAIRSPC**			
Collector	*I*	*E*_26_ − *E*_27_	-
Storage tank	*E*_26_ − *E*_27_	*E*_24_ − *E*_25_	-
Generator	*E*_24_ − *E*_25_	*E*_1_ + *E*_8_ − *E*_7_	-
Solution heat exchange	*E*_8_ − *E*_9_	*E*_7_ − *E*_6_	-
Pump	*W_sp_*	*E*_6_ − *E*_5_	-
Compressor	*W_com_*	*E*_11_ − *E*_14_	-
Cooling set	*E*_1_ + *E*_9_ + *E*_11_ − *E*_5_ − *E*_14_	*E*_15_ − *E*_17_	*E*_19_ − *E*_18_ + *E*_21_ − *E*_20_ + *E*_23_ − *E*_22_
Overall cooling system	*E*_24_ − *E*_25_ + *W_sp_* + *W_com_*	*E*_15_ − *E*_17_	*E*_19_ − *E*_18_ + *E*_21_ − *E*_20_ + *E*_23_ − *E*_22_
**SASCHCS**			
Collector	*I*	*E*_26_ − *E*_27_	-
Storage tank	*E*_26_ − *E*_27_	*E*_24_ − *E*_25_	-
Generator	*E*_24_ − *E*_25_	*E*_1_ + *E*_8_ − *E*_7_	-
Solution heat exchange	*E*_8_ − *E*_9_	*E*_7_ − *E*_6_	-
Pump	*W_sp_*	*E*_6_ − *E*_5_	-
Compressor	*W_com_*	*E*_11_ − *E*_15_	-
Cooling set	*E*_1_ + *E*_9_ + *E*_11_ − *E*_5_ − *E*_15_	*E*_16_ − *E*_17_	*E*_19_ − *E*_18_ + *E*_21_ − *E*_20_ + *E*_23_ − *E*_22_
Overall cooling system	*E*_24_ − *E*_25_ + *W_sp_* + *W_com_*	*E*_16_ − *E*_17_	*E*_18_+*E*_21_ − *E*_20_+*E*_23_ − *E*_22_

**Table 3:** Parameters of the investment cost.

*A_R_* = 100 m^2^, *W_R,SP_* = 10 kW, *W_R,COM_* = 100 kW	
Component	Value ($)
Generator	17,500
Condenser	8000
Evaporator/Subcooler	16,000
Absorber	16,500
Solution heat exchange	12,000
Solution pump	2100
Compressor	12,000
Valve	300

**Table 4:** Auxiliary equations of the cost flow rate for different components in the three systems.

Component	SNGDAC	SVCAIRSPC	SASCHCS
Generator	$\frac{C_{1}-C_{7}}{E_{1}-E_{7}}=\frac{C_{8}-C_{7}}{E_{8}-E_{7}}$	$\frac{C_{1}-C_{7}}{E_{1}-E_{7}}=\frac{C_{8}-C_{7}}{E_{8}-E_{7}}$	$\frac{C_{1}-C_{7}}{E_{1}-E_{7}}=\frac{C_{8}-C_{7}}{E_{8}-E_{7}}$
Solution heat exchange	$C_{8}/E_{8}=C_{9}/E_{9}$	$C_{8}/E_{8}=C_{9}/E_{9}$	$C_{8}/E_{8}=C_{9}/E_{9}$
Absorber (include the valve)	$\frac{C_{4}+C_{10}}{E_{4}+E_{10}}=\frac{C_{5}}{E_{5}}$	$\frac{C_{4}+C_{10}}{E_{4}+E_{10}}=\frac{C_{5}}{E_{5}}$	$\frac{C_{4}+C_{10}}{E_{4}+E_{10}}=\frac{C_{5}}{E_{5}}$
	$C_{9}/E_{9}=C_{10}/E_{10}$	$C_{9}/E_{9}=C_{10}/E_{10}$	$C_{9}/E_{9}=C_{10}/E_{10}$
Evaporator_1_/Subcooler	$C_{3}/E_{3}=C_{4}/E_{4}$	$C_{3}/E_{3}=C_{4}/E_{4}$	$C_{3}/E_{3}=C_{4}/E_{4}$
(include the valve_1_)	$C_{2}/E_{2}=C_{3}/E_{3}$	$C_{2}/E_{2}=C_{3}/E_{3}$	$C_{2}/E_{2}=C_{3}/E_{3}$
Condenser_1_	$C_{1}/E_{1}=C_{2}/E_{2}$	$C_{1}/E_{1}=C_{2}/E_{2}$	$C_{1}/E_{1}=C_{2}/E_{2}$
Evaporator_2_	—	$C_{13}/E_{13}=C_{14}/E_{14}$	$C_{11}/E_{11}=C_{12}/E_{12}$
(include the valve_2_)	—	$C_{12}/E_{12}=C_{13}/E_{13}$	$C_{14}/E_{14}=C_{15}/E_{15}$

**Table 5:** Comparisons of performance data of SVCAIRSPC with Reference [4].

Item	Model	Reference [4]	Difference (%)
Heat load of condenser of absorption subsystem (kW)	90.66	90.65	0.01
Heat load of condenser of compression subsystem (kW)	107.04	107.02	0.02
Heat load of absorber (kW)	105.83	108.52	2.48
Heat load of generator (kW)	111.49	114.18	2.36
Work of compressor (kW)	22.04	22.02	0.09
COP of absorption subsystem	0.762	0.744	2.42
COP of compression subsystem	3.856	3.86	0.10

**Table 6:** Design conditions of the three systems.

Parameters	SNGDAC	SVCAIRSPC	SASCHCS
Maximal installation area of collector array (m^2^)	270	270	270
Volume of storage tank (m^3^)	1.8	1.8	1.8
Inlet/outlet temperature of hot water in evacuated tube collectors (ETC) (°C)	100/105	100/105	100/105
Inlet/outlet temperature of hot water in storage tank (°C)	100/90	100/90	100/90
Effectiveness of solution heat exchange	0.7	0.7	0.7
Isentropic efficiency of compressor	0.7	0.7	0.7
Efficiency of solution pump	0.95	0.95	0.95
Inlet/outlet temperature of cooling water in condenser_1_ (°C)	32/37	32/37	32/37
Inlet/outlet temperature of cooling water in absorber (°C)	32/37	32/37	32/37
Inlet/outlet temperature of chilled water in evaporator_1_ (°C)	12/7	12/-	-
Degree of overlap in subcooler (°C)	-	-	9
Generator temperature of absorption system (°C)	85	85	85
Condenser temperature of absorption system (°C)	40	40	40
Absorber temperature of absorption system (°C)	40	40	40
Evaporator temperature of absorption system (°C)	5	-	5
Inlet/outlet temperature of cooling water in condenser_2_ (°C)	-	32/37	32/37
Inlet/outlet temperature of chilled water in evaporator_2_ (°C)	-	-/7	12/7
Condenser temperature of compression subsystem (°C)	-	40	40
Evaporator temperature of compression subsystem (°C)	-	-	5

**Table 7:** Summer cooling demands of office buildings located in Guangzhou and Turpan.

Number of Floors		1	2	3	4	5	6	7	8	9	10
Cooling demand (kW)	Guangzhou	47	111	158	204	251	197	344	390	437	483
	Turpan	48	115	162	208	255	301	348	394	441	487

**Table 8:** State property of the three chillers at base case (*Q_total_* = 158 kW).

point	*p* (kpa)	*T* (℃)	*m* (kg/s)	*x*	*e* (kJ/kg)	*c* ($/GJ)
**SNGDAC**						
1	7.326	85	0.0674	-	126.32	204.101
2	7.326	40	0.0674	-	1.435	204.101
3	0.864	5	0.0674	-	−7.699	204.101
4	0.864	5	0.0674	-	−176.39	204.101
5	0.864	40	2.941	0.579	42.126	118.129
6	7.326	40	2.941	0.579	42.133	118.189
7	7.326	70.5	2.941	0.579	47.559	125.887
8	7.326	85	2.873	0.592	50.468	124.898
9	7.326	53.5	2.873	0.592	52.542	124.898
10	0.864	53.5	2.873	0.592	52.542	124.898
**SVCAIRSPC**						
1	7.326	85	0.0139	-	126.32	60.124
2	7.326	40	0.0139	-	1.435	60.124
3	1.134	9	0.0139	-	−5.625	60.124
4	1.134	9	0.0139	-	−139.4	60.124
5	1.134	40	0.224	0.558	29.73	37.311
6	7.326	40	0.224	0.558	29.73	37.582
7	7.326	67.45	0.224	0.558	34.85	42.273
8	7.326	85	0.238	0.592	60.47	42.512
9	7.326	53.5	0.238	0.592	52.54	42.512
10	1.134	53.5	0.238	0.592	52.54	42.512
11	2416.84	63.5	0.8	-	−102.648	68.724
12	2416.84	40	0.8	-	−112.888	68.724
13	931.93	5	0.8	-	−118.048	66.632
14	931.93	5	0.8	-	−129.299	66.632
**SASCHCS**						
1	7.326	85	0.0136	-	126.32	59.531
2	7.326	40	0.0136	-	1.435	59.531
3	1.071	8.1	0.0136	-	−6.037	59.531
4	1.071	8.1	0.0136	-	−147.267	59.531
5	1.071	40	0.256	0.562	32.405	36.172
6	7.326	40	0.256	0.562	32.409	36.245
7	7.326	68.94	0.256	0.562	37.587	40.966
8	7.326	85	0.269	0.592	60.468	39.656
9	7.326	53.5	0.269	0.592	52.542	39.656
10	1.071	53.5	0.269	0.592	52.542	39.656
11	2416.84	63.5	0.805	-	−102.648	66.405
12	2416.84	40	0.805	-	−112.888	66.405
13	2416.84	17.1	0.805	-	−113.923	64.563
14	931.93	5	0.805	-	−115.193	64.563
15	931.93	5	0.805	-	−129.299	64.563

**Table 9:** Exergy analysis results of the three chillers at base case (*Q_total_* = 158 kW).

Component	*E_F_* (kW)	*E_P_* (kW)	*E_L_* (kW)	*E_D_* (kW)	*η_ex_*	*Y_D_*	*Y_L_*	Y_D_^*^ (%)
**SNGDAC**								
Generator	50.76	42.37	0.00	8.36	0.84	0.17	0.00	28.85
SHE	22.77	15.95	0.00	6.82	0.70	0.13	0.00	23.52
Pump	0.03	0.03	0.00	0.00	1.00	0.00	0.00	0.00
Cooling set	35.66	8.67	13.12	13.81	0.24	0.27	0.26	47.63
System	50.78	8.67	13.12	28.99	0.17	0.58	0.26	100.00
**SVCAIRSPC**								
Generator	8.65	7.06	0.00	1.60	0.82	0.04	0.00	5.56
SHE	1.79	1.23	0.00	0.56	0.69	0.01	0.00	1.96
Pump	0.00	0.00	0.00	0.00	1.00	0.00	0.00	0.00
Compressor	37.12	26.90	0.00	10.23	0.73	0.22	0.00	35.69
Cooling set	30.50	8.67	8.55	13.27	0.28	0.36	0.19	56.80
System	42.88	8.67	8.55	25.66	0.20	0.63	0.19	100.00
**SASCHCS**								
Generator	8.60	7.06	0.00	1.53	0.82	0.04	0.00	6.87
SHE	2.21	1.40	0.00	0.63	0.69	0.02	0.00	2.83
Pump	0.00	0.00	0.00	0.00	1.00	0.00	0.00	0.00
Compressor	29.60	21.45	0.00	8.16	0.73	0.21	0.00	36.51
Cooling set	27.88	8.67	7.19	12.02	0.31	0.32	0.19	53.80
System	38.20	8.67	7.19	22.34	0.23	0.59	0.19	100.00

**Table 10:** Exergoeconomic analysis results of the three chillers at base case (*Q_total_* = 158 kW).

Component	*c_F_* ($/GJ)	*c_P_* ($/GJ)	*C_D_* ($/yr)	*C_L_* ($/yr)	*Z* ($/yr)	*C_D_* + *C_L_* + *Z* ($/yr)	*r*	*f*
**SNGDAC**								
Generator	108.783	132.940	6878.945	0.000	863.840	7742.785	0.222	0.116
SHE	121.104	178.145	6242.720	0.000	637.041	6879.761	0.471	0.093
Pump	38.056	323.226	0.000	0.000	49.145	49.145	7.494	1.000
Cooling set	160.890	280.685	16795.81	15956.5	2118.187	34654.537	0.745	0.061
System	108.751	280.685	29917.48	15956.54	3668.212	49326.23	1.581	0.074
**SVCAIRSPC**								
Generator	30.46	41.71	366.94	0.00	233.13	600.07	0.37	0.39
SHE	39.35	69.15	166.83	0.00	109.89	276.72	0.76	0.40
Pump	38.06	605.53	0.00	0.00	4.13	4.13	14.91	1.00
Compressor	38.06	56.62	2942.00	0.00	832.05	3774.05	0.49	0.22
Cooling set	54.87	133.76	6750.36	3503.97	1597.92	11852.26	1.44	0.14
System	36.62	133.76	10226.13	3503.97	2777.12	16507.22	2.65	0.11
**SASCHCS**								
Generator	30.471	41.496	353.251	0.000	235.433	588.684	0.362	0.400
SHE	38.784	67.082	185.530	0.000	112.969	298.499	0.730	0.378
Pump	38.055	556.774	0.000	0.000	4.258	4.258	13.63	1.000
Compressor	38.055	56.617	2346.149	0.000	663.534	3009.683	0.488	0.220
Cooling set	54.627	104.676	4963.080	2968.227	1579.368	9510.675	0.916	0.166
System	36.349	104.676	7848.009	2968.227	2595.561	13411.797	1.880	0.194

## Nomenclature

*A*	area (m^2^)
*Ar*	Archimedes number based on tube diameter
*c*	cost per exergy unit ($/GJ)
*c_p_*	specific heat (kJ/kg·°C)
*C*	cost flow rate ($/s)
*CEPCI*	chemical engineering plant cost index
*COP*	coefficient of performance
*CRF*	capital recovery factor
*e*	specific exergy (kJ/kg)
*E*	exergy rate (kW)
*d*	diameter (m)
*F*	fouling factor (m^2^·°C/kW)
*g*	acceleration of gravity (m/s^2^)
*h*	specific enthalpy (kJ/kg), heat transfer coefficient (W/m^2^·°C)
*i*	Chinese interest rate
*I*	solar irradiance (W/m^2^)
*LMTD*	logarithmic mean temperature difference
*m*	mass flow rate (kg/s)
*N*	operational period
*Nu*	Nusselt number
*Pr*	Prandtl number
*Q*	heat flux (kW/m^2^), heat load (kW)
*r*	latent heat (kJ/kg)
*Re*	Reynolds number
*s*	specific entropy (kJ/kg)
*T*	temperature (°C)
*U*	heat transfer coefficient
*W*	work (kW)
*We*	Weber number
*x*	concentration of solution
*X*	Lockhart-Martinelli parameter
*Z*	investment cost

## Greek symbols

*ρ*	density (kg/m^3^)
*η*	efficiency
*λ*	thermal conductivity (kW/m·°C)
*μ*	viscosity (kg/m^3^)
*σ*	surface tension (N/m)
*Г*	mass flow rate per wetted perimeter (kg/m^3^)
*ε_n_*	correction factor of tube bundle

## Subscripts

*0*	environmental state
*a*	surrounding, absorption chiller
*COM*	compressor
*col*	collector
*D*	exergy destruction
*ex*	exergy
*E*	evaporator
*etc*	evacuated tube collector
*F*	fuel
*g*	generator
*HE*	heat exchanger
*hyb*	hybrid system
*i*	inlet
*l*	liquid
*L*	loss
*o*	Outlet
*oy*	original year
*pool*	pool boiling
*P*	product
*R*	reference state
*s*	isentropic
*sat*	saturation
*sys*	system
*SP*	solution pump
*ST*	storage tank
*v*	vapour
*w*	wall
